# Peer Review of “Cardiotoxicity in Pediatric Cancer Survivorship: Retrospective Cohort Study”

**DOI:** 10.2196/79523

**Published:** 2025-07-31

**Authors:** John Lucas Jr

**Affiliations:** 1 St. Jude Children's Research Hospital, Memphis, TN, United States

**Keywords:** cardiotoxicity, cardiology, cardiovascular, heart, arrhythmias, self-reported questionnaires, oncology, survivors, pediatrics, prevalence, incidence, risk, epidemiology, anthracycline exposure, childhood cancer survivors

*This is a peer-review report for “Cardiotoxicity in Pediatric Cancer Survivorship: Retrospective Cohort Study.”*

## Round 1 Review

### Overall Evaluation

This significant and timely manuscript [1], which investigates the long-term cardiovascular complications in pediatric cancer survivors, has notable strengths, including its large cohort size, long-term follow-up, and utilization of a well-established dataset (Childhood Cancer Survivor Study). The methodology is generally sound, and the findings contribute meaningfully to our understanding of cardiotoxicity risks in childhood cancer survivors. However, certain areas necessitate clarification and additional analyses. These are detailed below.

The study relies heavily on self-reported cardiovascular complications, which may introduce reporting bias. While a subset of cases was validated via medical records, the proportion of validated cases is not explicitly stated, and the possibility of underreporting or overreporting remains. The reliance on self-reported cardiovascular complications may have introduced reporting bias into the study. Although some cases were validated through medical records, the proportion of validated cases remains unclear, leaving the potential for underreporting or overreporting. The authors could also consider exploring linkage with external databases (eg, insurance claims, hospital records) for additional validation.

The manuscript presents a risk prediction model (C statistic 0.78), but there is no external validation or discussion of its clinical applicability. Validate the model using an independent dataset (eg, a subset of Childhood Cancer Survivor Study data withheld from model training or another survivor cohort). Report calibration metrics (eg, Hosmer-Lemeshow test, calibration plots) to assess model accuracy. Provide a clinical risk score or decision framework for practical implementation.

The study reports a decreasing risk of cardiotoxicity over time, suggesting improvements in treatment protocols. However, this could be confounded by survivor selection bias (eg, patients with higher early mortality due to severe toxicity were less likely to be included in later eras).

Adjust for potential survivor bias using inverse probability weighting or sensitivity analyses. Consider comparing treatment regimens (eg, changes in anthracycline dosages, cardioprotective measures) across eras to explicitly determine which interventions contributed to reduced risk. The research indicates that the risk of cardiotoxicity diminishes over time, suggesting that treatment protocols have become more effective. However, it is possible that this observation is attributable to survivor selection bias, wherein patients who succumbed to severe toxicity early in the study were not included in subsequent phases. To address potential survivor bias, researchers should employ methodologies such as inverse probability weighting or sensitivity analyses. Additionally, treatment regimens (eg, modifications in anthracycline dosages and cardioprotective measures) should be compared across different time periods to ascertain which interventions are responsible for the diminished risk.

The study focuses on clinically evident cardiovascular complications but does not assess subclinical cardiotoxicity, which could be detected via biomarkers or imaging.

Incorporate cardiac biomarkers (eg, troponins, N-terminal pro–brain natriuretic peptide) in a subset of survivors to identify early signs of myocardial damage. Perform echocardiographic or cardiac magnetic resonance imaging evaluations in a subgroup to detect preclinical cardiac dysfunction. This could strengthen the study’s ability to recommend early intervention strategies.

The authors appropriately point out the opportunity to improve early intervention by identifying a subset of survivors for early myocardial damage using cardiac biomarkers and imaging. While this is not possible in the present study, future studies incorporating this approach would allow for detection of subclinical cardiotoxicity.

The manuscript discusses risk factors but does not evaluate protective factors (eg, exercise, angiotensin-converting enzyme inhibitors, β-blockers). Analyze whether lifestyle modifications (eg, regular exercise) or cardioprotective medications influence the incidence of cardiotoxicity. Conduct a subgroup analysis on survivors who received cardioprotective interventions versus those who did not.

Please indicate whether the proportional hazards assumptions were tested and consider reporting Schoenfeld residuals or time-dependent covariate analyses.

Please include more details on how missing data were handled.

Were there particular domains of quality of life that were lower among those with cardiovascular complications?

Consider adding detailed figure legends to improve readability and refining axis labels in existing figures.

A table summarizing key risk factors with adjusted hazard ratios and *P* values would be beneficial.

## Round 2 Review

Please state the proportion of cases with cardiovascular events confirmed by medical record review.

Please discuss the increased cardiotoxicity observed in male survivors. Was this due to treatment or other comorbidities that exacerbated previously subclinical cardiac exposures?

Please provide a thoughtful description of how the risk model could be integrated into previously described models and recommendations for cardiac risk groups like the International Late Effects of Childhood Cancer Guideline Harmonization Group.

Please standardize the reporting/formatting for data into a table format more typical for manuscript reporting for complication rates, multivariate cox regression, and temporal trends.

Please provide a table or figure for the treatment era analysis.

Please provide a table or figure for the sibling controls comparison. Is this after adjustment for age, gender, etc?

The CI of cardiovascular complications in childhood cancer survivors data is shown in a nonstandard stacked bar plot format. Please show as CI curves.
